# Antiemetic and Myeloprotective Effects of* Rhus verniciflua Stoke* in a Cisplatin-Induced Rat Model

**DOI:** 10.1155/2017/9830342

**Published:** 2017-02-08

**Authors:** Hyo-Seon Kim, Hyeong-Geug Kim, Hwi-Jin Im, Jin-Seok Lee, Sung-Bae Lee, Won-Yong Kim, Hye-Won Lee, Sam-Keun Lee, Chang Kyu Byun, Chang-Gue Son

**Affiliations:** ^1^Liver and Immunology Research Center, Daejeon Oriental Hospital of Daejeon University, 176-9 Daeheung-ro, Jung-gu, Daejeon 34929, Republic of Korea; ^2^TKM-Based Herbal Drug Research Group, Korea Institute of Oriental Medicine, Daejeon 34052, Republic of Korea; ^3^Department of Applied Chemistry, Daejeon University, 62 Daehak-ro, Dong-gu, Daejeon 34520, Republic of Korea

## Abstract

*Rhus verniciflua* Stoke has been commonly used in traditional medicine to treat gastrointestinal (GI) dysfunction diseases. In order to investigate pharmacological properties of* Rhus verniciflua* Stoke water extract (RVX) on cisplatin-induced amnesia, RVX (0, 25, 50, or 100 mg/kg) was orally administrated for five consecutive days after a single intraperitoneal injection of cisplatin (6 mg/kg) to SD rat. Cisplatin injection significantly increased the kaolin intake (emesis) but reduced the normal diet intake (anorexia) whereas the RVX treatment significantly improved these abnormal diet behaviors at both the acute and delayed phase. The serotonin concentration and the related gene expressions (5-HT3 receptors and SERT) in small intestine tissue were abnormally altered by cisplatin injection, which were significantly attenuated by the RVX treatment. Histological findings of gastrointestinal tracts, as well as the proteins level of proinflammatory cytokines (TNF-*α*, IL-6, and IL-1*β*), revealed the beneficial effect of RVX on cisplatin-induced gastrointestinal inflammation. In addition, RVX significantly improved cisplatin-induced myelosuppression, as evidenced by the observation of leukopenia and by histological examinations in bone marrow. Our findings collectively indicated* Rhus verniciflua* Stoke improved the resistance of rats to chemotherapy-related adverse effects in the gastrointestinal track and bone marrow.

## 1. Introduction

A recent study reported that more than 14.1 million people per year are newly diagnosed with various malignancies worldwide [1]. The application of anticancer therapies depends on the status of clinical progression, characteristics of tumors, and patient' conditions [2]. Regarding treatments for patients with cancers, various medical modalities, including surgery, radiotherapy, chemotherapy, and immunotherapy, have been developed [3, 4]. Especially for patients with advanced cancers, chemotherapy is the first choice for more than 8 million patients annually in the United States [5, 6]. Chemotherapy, however, inevitably induces diverse adverse effects including emetic symptoms, leukopenia, gastrointestinal toxicity, hair loss, and fatigue [7]. Generally, 70 to 80% of patients undergoing chemotherapy complain mainly of the emetic symptoms, such as vomiting and nausea [8, 9]. Conventional agents for emesis include 5-hydroxytryptamine 3 (5-HT3) receptor antagonists, neurokinin 1 (NK1) receptor antagonists, glucocorticoids, and metoclopramide [10]. However, these drugs are only effective in either acute emesis or delayed emesis [11], or they evoke additional adverse effects, including insomnia, constipation, diarrhea, and headache [12, 13].

On the other hand, herbal plants or their derived natural compounds have attracted increasing attention in anticancer drug studies particularly regarding antiadverse effects, such as the emesis which was evoked by gastro enteric dysfunctions.* R. verniciflua* Stoke, called the lacquer tree, belonging to the Anacardiaceae family, has traditionally been described for treating digestive troubles [14]. According to the traditional Chinese medical literature, known as* Ben-cao-gang-mu* (本*草纲*目), the pharmacological efficacies of* R. verniciflua* on the gastrointestinal tract problems have been well indicated [15].

Several animal studies have reported that* R. verniciflua* exerted beneficial effects on inflammation and several metabolic diseases [16, 17] and protective effects against drug toxicity [18]. The previous studies suggested that* R. verniciflua* would be a potent candidate for drug development to treat chemotherapy-induced side effects, particularly emesis.

The present study therefore aimed to investigate the effects of* R. verniciflua* water extract (RVX) against chemotherapy-related adverse effects, particularly emesis and gastrointestinal inflammation using a model of rats injected with cisplatin.

## 2. Methods

### 2.1. Procedure for Fingerprinting and Sequencing Analysis

*R. verniciflua* was obtained from a local specific farm for* R. verniciflua* in Ok-Cheon (Chung-buk, South Korea). After obtaining* R. verniciflua* water extract (RVX), a final yield of 0.70% (w/w), we conducted fingerprint using a HPLC-DAD instrument (YL9100 series, South Korea) as described in Supplementary Information (see Supplementary Material available online at https://doi.org/10.1155/2017/9830342).

Genomic DNA from the leaves of RVX was extracted using a DNeasy Plant Mini kit (Qiagen, Valencia, CA, United States). The internal transcribed spacer (ITS) gene, including 5.8s ribosomal DNA, was amplified by polymerase chain reaction (PCR) using the primers for ITS1 (5′-TAG CGC AGA ACG ACC CGC CAA CCT GTA T-3′) and ITS2 (5′-CAC CTG ACC TGG GGT CGC GAT GCG-3′). Amplification of ITS fragments was conducted with the 2X SG Taq Master Mix (LPS solution, Daejeon, South Korea), and PCR conditions were obtained using an IQ5 PCR Thermal Cycler (Bio-Rad, Hercules, CA, United States) in the following steps: predenaturation at 95°C for 5 min, 35 cycles of 95°C/30 s (denaturation), 55°C/40 s (annealing), 72°C/1 min (extension), and final extension at 72°C for 5 min. The PCR products were analyzed on 1% agarose gel and distinguishable bands were purified using QIAQuick Gel extraction kit (Qiagen). The products were sequenced using an BigDye Terminator v3.1 cycle sequencing kit (Applied Biosystems, CA, United States) with an ABI3730XL (Applied Biosystems) automated sequencer. After alignment by ClustalW, the results were analyzed using BioEdit 7.0 version and vector NTI advance software, version 11.

### 2.2. Chemical Materials

The reagents for the present study were as follows: cisplatin (cis-diammineplatinum (II) dichloride), metoclopramide, protocatechuic acid, fustin, fisetin, sulfuretin, and butein were obtained from Sigma (St. Louis, MO, United States). Arabic gum was purchased from Junsei Chemical Co., Ltd. (Tokyo, Japan). Calci-Clear Rapid was purchased from National Diagnostics (Atlanta, GA, United States). Olive oil was purchased from DC Chemical Co., Ltd. (Seoul, South Korea).

### 2.3. Kaolin Diet Preparation

Kaolin (H_2_Al_2_Si_2_O_8_·H_2_O) was prepared according to a previous method [19]. Briefly, pharmacological-grade kaolin (Samchun Pure Chemical Co., Ltd., Pyeongtaek, South Korea) was mixed with 1% acacia or gum Arabic in double-distilled water to form a thick paste. The paste was rolled and cut into small pellets. Pellets were dried completely at room temperature for 3-4 days and were maintained in sterile conditions.

### 2.4. Animals and Experimental Design

A total of 36 specific pathogen-free Sprague-Dawley male rats (6 weeks old, 160–180 g) were purchased from Dae-Han Bio Link (Chung-buk, South Korea). The rats were housed in a controlled temperature room at 22 ± 2°C, in 55%  ±  10% relative humidity with a 12 h : 12 h light-dark cycle, and they were freely fed commercial standard chow (Dae-Han Bio Link) and were provided tap water ad libitum for 7 days. After acclimation, the rats were housed separately in cages and were familiarized with the testing procedures. In addition to routine rat chow and tap water, a measured quantity of kaolin (5 g) pellets was provided in separate containers for 3 days prior to the experiment to allow the rats to adapt to its presence psychologically. After 3 days of habituation, the animals were subjected to pica experiments.

Acclimatized rats were divided into six groups (*n* = 6): normal and control groups, three dosages of RVX treatment groups (25, 50, or 100), and a Met 25 group. At 0 h, the normal group received saline, while all of the other groups received a single intraperitoneal injection of cisplatin in saline (6 mg/kg). Two hours after cisplatin injection, each group was initially treated with oral administration of water (normal and control groups), RVX (25, 50 or 100 mg/kg), or metoclopramide (25 mg/kg), followed by the same administration at 24 h intervals for 96 h. All of the groups were allowed free access to kaolin diets until 120 h. All of the rats were monitored for their consumption of normal and kaolin diets, and their body weights were measured daily. On the last experimental day, all rats were sacrificed by collection of whole blood via the abdominal vein under ether anesthesia.

This animal experiment was approved by the Institutional Animal Care and Use Committee of Daejeon University (DJUARB2015-041) and was conducted in accordance with the Guide for the Care and Use of Laboratory Animals published by the US National Institutes of Health (Bethesda, MD, United States).

### 2.5. Hematology and Histopathology

On the final day of the experiment, hematological parameters were measured using a HEMA VET 850 automatic analyzer (CDC Technologies, United States). The white blood cell (WBC), neutrophil, lymphocyte, monocyte, basophil, eosinophil, red blood cell (RBC), hemoglobin, and platelet counts were determined. The spleen and thymus were removed, and their weights were measured. The stomach tissue, small intestine (both proximal and distal), and colon were dissected, washed in ice-cold phosphate buffered saline, and fixed in 10% formalin for 3 days. The paraffin-embedded samples were sectioned (4 *μ*m thickness) and slides stained with H&E or Masson's trichrome staining. Bone marrow was decalcified with Calci-Clear Rapid (National Diagnostics), then embedded in paraffin, sectioned at 6 *μ*m, and stained with H&E stain. Representative images were obtained using a light microscope (Leica Microsystems, Wetzlar, Germany). The percentage areas of positively stained cells were analyzed using Image J image analysis software (Rasband, Bethesda, MD, United States), version 1.46.

### 2.6. Quantitative Real-Time PCR Analysis

Total RNA was extracted from small intestine samples using Trizol reagent (Molecular Research Center, Cincinnati, OH, United States). cDNA was synthesized from total RNA (2 *μ*g) in a 20 *μ*L reaction using the High-Capacity cDNA reverse transcription kit (Ambion, Austin, TX, United States). Real-time PCR was performed using SYBR Green PCR Master Mix (Applied Biosystems, Foster City, CA, United States) with PCR amplification performed in accordance with a standard protocol using the IQ5 PCR Thermal Cycler (Bio-Rad, Hercules, CA, United States). The following primers were used (5′→3′, forward and reverse): 5-HT3A receptor, GGA CTC CTG AGG ACT TCG ACA A and TTC CCC ACG TCC ACA AAC TC; SERT, CTG TTC ATC ATT TGC AGT TTT CTG A and TCC CTA TGC AGT AGC CCA AGA; and *β*-actin, AGG CCA ACC GTG AAA AGA TG and CCA GAG GCA TAC AGG GAC AAC.

### 2.7. Measurement of Serotonin and Proinflammatory Cytokines

Serotonin in the small intestine was measured using a serotonin ELISA kit (LDN, Nordhorn, Germany) and stomach tissue levels of proinflammatory cytokines were analyzed using commercial ELISA kits (TNF-*α* from BD OptEIA, CA, United States; IL-6 and IL-1*β* from R&D Systems, MN, United States).

### 2.8. Statistical Analysis

The results are expressed as the means ± standard deviations (SD). The statistical significance of differences between groups was analyzed by one-way analysis of variance (ANOVA), followed by Fisher's least-significant difference (LSD) test, or Student's *t*-test. In all of the analyses, values of *p* < 0.05 were considered statistically significant.

## 3. Results

### 3.1. Fingerprint of RVX and Verification of* R. verniciflua*

**Figure 1:**
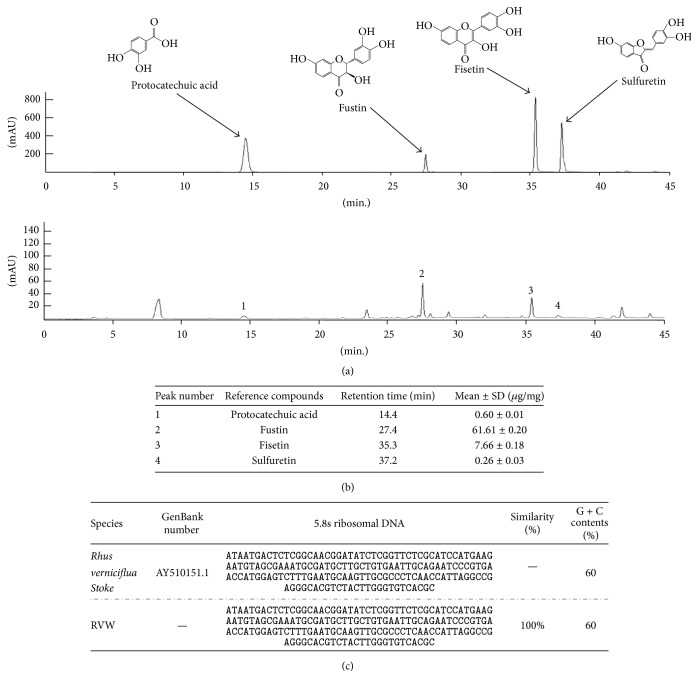
HPLC fingerprint and sequencing analysis. RVX and its standard compounds were subjected to HPLC. Chromatogram of reference compounds mixture and RVX (a). The quantitative analysis of each component in RVX (b). The comparative 5.8s rDNA sequences between* R. verniciflua* Stoke and RVX were exhibited (c).

The chemical constitution analysis of RVX was evaluated by performing HPLC-DAD analysis using four reference components: one phenolic compound (protocatechuic acid) and three flavonoid compounds (fustin, fisetin, and sulfuretin), respectively (Figure 1(a)). The fustin was quantified as the prevalent compound (61.61 ± 0.20 *μ*g/mg), and it was followed by fisetin, protocatechuic acid, and sulfuretin, respectively (Figure 1(b)). The gene sequences of the ITS1-5.8s-ITS2 region coincided completely with* R. verniciflua* Stoke (AY510151.1, GenBank) (“*Toxicodendron vernicifluum* internal transcribed spacer 1, 5.8S ribosomal RNA gene, and internal transcribed spacer 2, complete sequence,” 2004) (Figure 1(c)).

### 3.2. Changes in Kaolin Diet Intake

**Figure 2:**
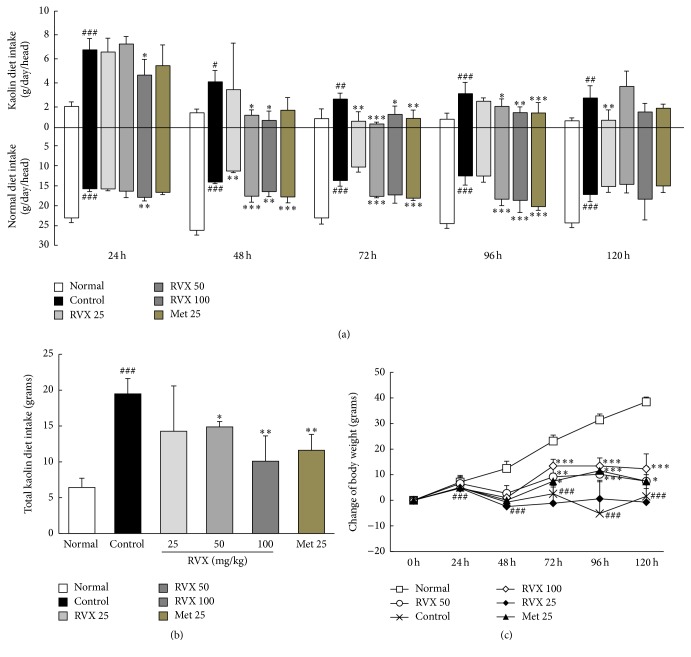
Kaolin consumption, normal diet consumption, and body weight change. The consumption of kaolin and a normal diet at different time points (a), total kaolin consumption (b), and body weight change (c) were monitored. Data are expressed as the mean ± SD (*n* = 6). ^#^*p* < 0.05, ^##^*p* < 0.01, and ^###^*p* < 0.001, compared with the normal group; ^*∗*^*p* < 0.05, ^*∗∗*^*p* < 0.01, and ^*∗∗∗*^*p* < 0.001, compared with the control group.

Cisplatin injection (6 mg/kg) considerably increased kaolin diet intake by 2.6- to 3.3-fold, between 24 h and 120 h, compared with the normal group. Kaolin diet intake was significantly decreased by RVX 25 mg/kg (at 72 h and 120 h, *p* < 0.01), RVX 50 mg/kg (from 48 h to 72 h, *p* < 0.05 or *p* < 0.001), and RVX 100 mg/kg (from 24 h to 96 h, *p* < 0.05 or *p* < 0.01, Figure 2(a)). Metoclopramide suppressed kaolin intake to a substantial extent from 72 h to 96 h. When the analysis was conducted on total kaolin intake over 0–120 h, the cisplatin-induced increase in total kaolin intake (3.0-fold) was significantly attenuated by RVX treatment (*p* < 0.05 for 50 mg/kg; *p* < 0.01 for 100 mg/kg, Figure 2(b)).

### 3.3. Changes of Normal Diet Intake and Body Weight

Cisplatin injection significantly reduced normal diet intake by 0.5- to 0.7-fold, between 24 and 120 h, compared with the normal group, while it was significantly attenuated by administration of RVX 50 mg/kg (from 48 to 96 h, *p* < 0.001) and RVX 100 mg/kg (from 24 to 96 h, *p* < 0.01 or *p* < 0.001, Figure 2(c)). Body weight was also significantly reduced by 47.14 g, compared with normal group (at 120 h, *p* < 0.001). Treatment with RVX (50 and 100 mg/kg), however, significantly ameliorated this weight loss compared with the control group (Figure 2(c)). Metoclopramide showed similar effects to RVX (50 mg/kg) in both normal diet intake and recovery of weight loss.

### 3.4. Histopathological Findings for Gastrointestinal Damage

**Figure 3:**
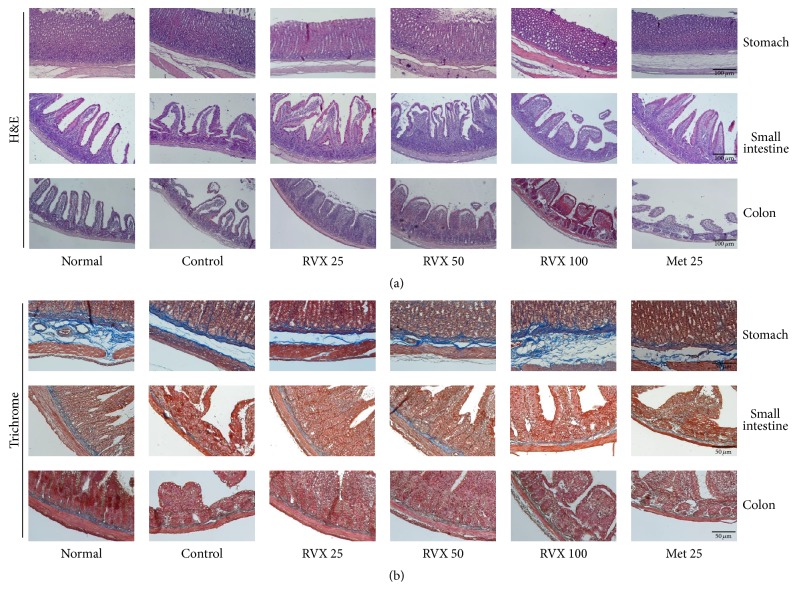
Histopathological findings of the gastrointestinal tract. H&E staining was conducted for the stomach, small intestine, and colon (a) and Masson's trichrome staining (b). The stained tissues were examined under a light microscope (200x magnification).

Hematoxylin and Eosin (H&E) staining revealed that cisplatin injection induced severe degenerative changes, especially in the small intestine and colon, as characterized by disruption of the epithelial architecture and dilated intercellular spaces. Treatment with RVX remarkably reversed these pathological alterations, compared with control group. In stomach tissue, no marked damage but intensive blue staining was observed, indicating migration of gastric chief cells to the outer layer of the stomach lining, in only control and metoclopramide groups but not in the RVX-treated groups (Figure 3(a)). Masson's trichrome staining confirmed loss of collagen at the submucosal region in the small intestine and colon of the control group, whereas RVX treatments notably reduced these collagen losses (Figure 3(b)).

### 3.5. Serotonin Concentrations and Related Gene Expressions in Small Intestine Tissue

**Figure 4:**
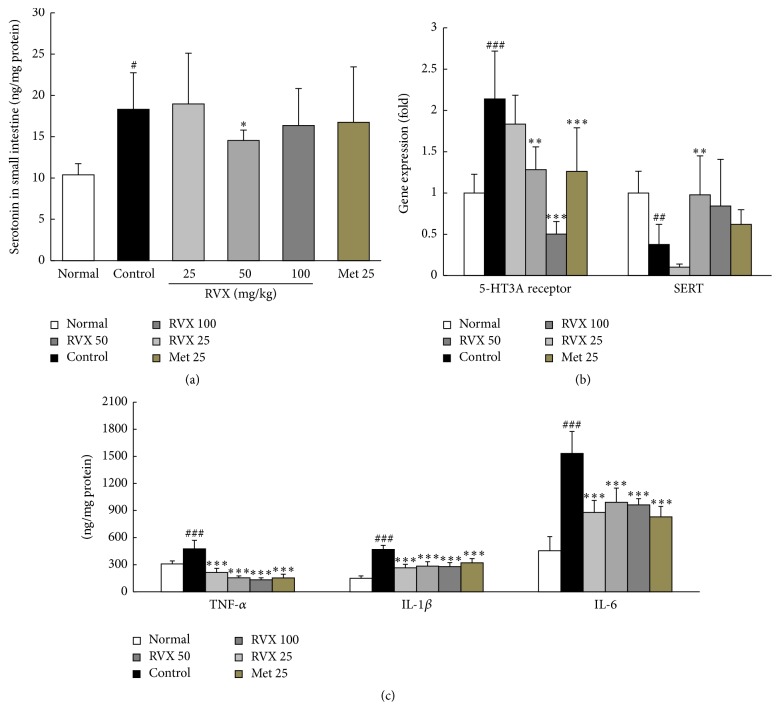
Serotonin and its related genes and proinflammatory cytokines. Serotonin concentrations in the small intestine were measured using ELISA (a). The gene expression levels of 5-HT3A receptor and SERT were measured using real-time PCR (b). Protein levels of TNF-*α*, IL-6, and IL-1*β* (c) were performed using ELISA method. Data are expressed as the mean ± SD (*n* = 6). ^#^*P* < 0.05, ^##^*p* < 0.01, and ^###^*p* < 0.001 compared with the normal group; ^*∗*^*p* < 0.05, ^*∗∗*^*p* < 0.01, and ^*∗∗∗*^*p* < 0.001 compared with the control group.

The concentration of serotonin in small intestine tissue was elevated by 1.8-fold by cisplatin injection compared with the normal group, while RVX treatment (especially 50 mg/kg) significantly attenuated this compared with the control group (*p* < 0.05, Figure 4(a)). Cisplatin injection significantly upregulated mRNA expression of 5-HT3A receptor (2.1-fold) but downregulated serotonin transporter (SERT, 0.5-fold) compared with the normal group in small intestine tissue (*p* < 0.001). Treatment with RVX significantly modulated these alterations of 5-HT3A receptor (*p* < 0.01 for 50 mg/kg, *p* < 0.001 for 100 mg/kg) and SERT (*p* < 0.01 for 50 mg/kg, *p* < 0.05 for 100 mg/kg) compared with the control group, respectively (Figure 4(b)). Metoclopramide showed similar effects of RVX (50 mg/kg) on the expression of 5-HT3A receptor, but not on the serotonin concentration or SERT gene expression.

### 3.6. Protein Levels of Proinflammatory Cytokines in Stomach Tissue

Cisplatin injection considerably elevated the protein levels of proinflammatory cytokines, including TNF-*α* (1.5-fold), IL-6 (3.4-fold), and IL-1*β* (3.4-fold) in stomach tissue, compared with the normal group. These abnormal elevations of the three cytokines were significantly attenuated by RVX treatment compared with the control group (*p* < 0.001, Figure 4(c)). Metoclopramide showed similar effects to RVX treatment.

### 3.7. Changes in Hematological Parameters

On the final day of the experiment, the control group showed the leukopenia in approximately 57% of the normal group; in particular, lymphocyte counts were decreased in half that of the normal group. These alterations were significantly improved to near to the normal group by RVX treatment (especially 100 mg/kg, *p* < 0.001, Table 1). The counts of red blood cells and platelets and hemoglobin concentration were not notably affected by cisplatin injection. The lymphocyte count was improved by moderate level in the metoclopramide group.

### 3.8. Histopathological Findings for Bone Marrow Damage

**Figure 5:**
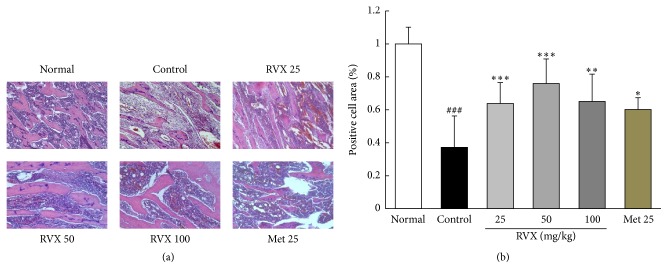
Histopathological finding of bone marrow. H&E staining was conducted for bone marrow, and the results were examined under a light microscope (200x magnification) (a). The positive cells area was quantitatively analyzed using Image J software, version 1.64 (b). Data are expressed as the mean ± SD (*n* = 6). ^###^*p* < 0.001 compared with the normal group; ^*∗*^*p* < 0.05, ^*∗∗*^*p* < 0.01, and ^*∗∗∗*^*p* < 0.001 compared with control group.

H&E staining revealed that cisplatin injection considerably injured bone marrow tissue, as evidenced by loss of bone marrow cells and thinning of trabecular bone, compared with the normal group. These alterations (especially vacant spaces in bone marrow) notably attenuated by RVX treatment compared with the control group (*p* < 0.01 or 0.001, Figures 5(a) and 5(b)). Metoclopramide also improved those pathological alterations of bone marrow compared with the control group.

### 3.9. Changes of Spleen and Thymus Weights

Cisplatin injection considerably decreased the weights of both the thymus (0.5-fold) and spleen (0.6-fold), compared with the normal group; however, treatment with RVX (50 and 100 mg/kg) significantly attenuated these changes in organ weights (*p* < 0.05, 0.01, or 0.001) compared with the control group (Table 1). This positive finding was observed in the metoclopramide group, for thymus weight, but not spleen weight.

## 4. Discussion

*R. verniciflua* has been traditionally used to treat* “blood stasis* (*瘀血*)*”* which is associated with chronic diseases, including pain, stomach problem, and cancer, according to the theory of traditional Oriental medicine [14].* R. verniciflua* was described in the herbal formulae extract part of traditional Chinese medicine literature (*Ben-cao-gang-mu*) for ameliorating gastro enteric dysfunctions [15]. Besides,* R. verniciflua* has been used to treat digestive troubles by adding it to chicken soup in traditional Korean medicine [20, 21]. Regarding the adverse effect of chemotherapy, particularly emesis, the pharmacological mechanisms of* R. verniciflua* still remain unclear. Herein, thus, we evaluated the pharmaceutical effects of* R. verniciflua* against cisplatin-induced adverse effects, especially gastrointestinal distortion. Cisplatin is an anticancer drug of a platinum-containing class used to treat various types of cancers, including sarcomas, small cell lung cancer, ovarian cancer, and lymphomas, but it presents a number of side effects [22, 23]. Low reproducibility has frequently became an issue in pharmaceutical studies using herbal materials [24]. To minimize biased results due to poor quality of samples, we conducted fingerprint analysis and gene sequence-based vivification of the plant species,* R. verniciflua*.

As expected, a single injection of cisplatin remarkably increased the kaolin diet intake (increased emesis) and reduced the normal diet intake (increased anorexia) in the control group. Approximately 90% of the patients receiving cisplatin suffer from nausea and vomiting, and anorexia is also often accompanied by emesis in these patients [25]. This study adapted a pica model in which animals responded to emetic stimuli by consuming nonnutritive substances, such as kaolin [26]. The behavioral changes in food intake have frequently been assessed in animals incapable of vomiting [27]. In general, chemotherapy-induced emesis is divided into two phases: acute (24 h) and delayed (24–120 h), respectively [28], and RVX treatment ameliorated phases of emesis and anorexia.

The activation of 5-HT3 receptor with serotonin (5-HT) plays a key role in chemotherapy-induced-emesis [29]. Chemotherapy can induce 5-HT release from the enterochromaffin (EC) cells, consequently stimulating 5-HT3 receptor on the vagal nerve and resulting in the activation of the vomiting reflex [30]. As expected, RVX treatment significantly improved the cisplatin-induced upregulation of 5-HT3 receptor gene expression as well as the activated release of 5-HT in the intestine. Intestinal 5-HT is transported via SERT and then is metabolized in the epithelial cells of the gastrointestinal tract; therefore, damage to epithelial cells can impair the 5-HT metabolism, leading to prolonged contact time of 5-HT with 5-HT3 receptor [31]. RVX treatment normalized the downregulated gene expression of SERT in the intestine after cisplatin injection. Metoclopramide 25 mg/kg exerted antiemetic effects in only delayed phase, and it affected the gene expression of 5-HT3 receptor but not of SERT.

Histopathological findings showed severe gastrointestinal tract damage by cisplatin injection in our study, as evidenced by remarkable loss of villus cells, disruption of the epithelial architecture, and considerable reduction in collagen contents via H&E staining as well as trichrome staining. These alterations were more notable in the small intestine and colon than stomach; however, the stomach showed the intensive nuclei in a blue color, supposedly because of migration of gastric chief cells to the outer layer of the stomach lining. Gastric chief cells stain basophilic upon H&E staining owing to the large proportion of rough endoplasmic reticulum in its cytoplasm. These cells are generally located deep in the mucosal layer of the stomach lining; the gastric chief cells then move into outer layer in response to inflammation [32]. This alteration was notably attenuated in groups treated with RVX, but not metoclopramide treatment. Chemotherapy treatment with cisplatin is also well known for provoking the gastritis [33]. While proinflammatory cytokines, including TNF-*α*, IL-6, and IL-1*β*, were remarkably elevated in stomach tissue, these abnormal elevations of these three cytokines were significantly improved by RVX treatment in our results.

On the other hand, bone marrow is one of the most fragile organs during chemotherapy. Myelosuppression increases the risk of infection, fatigue, and diminished quality of life (QoL) in patients; thus, it is a major dose-limiting factor for the clinical use of chemotherapy [34]. Moreover, myelosuppression, for instance, leukopenia, is primarily the reason for the delay, reduction, or cessation of chemotherapy treatment, occurring in approximately 15% of total cases of chemotherapy [35]. Our previous study reported that the most severe leukopenia was observed at 5 days after a single cisplatin injection rat model [36]. As we expected, notable bone marrow suppression and leukopenia were observed 5 days after cisplatin injection, and these pathologic conditions were considerably reserved by RVX treatment, especially at the highest dose of 100 mg/kg. In addition to the bone marrow, the spleen and thymus are representative immune organs, and losses in their weights in experimental models of cisplatin injection have been widely reported [37], significantly attenuated by RVX treatment in our current study. These findings anticipated that RVX may have potential for use against immunosuppression caused by chemotherapy. The current results are in accordance with previous study, which RVS ameliorated significantly the cisplatin-induced adverse effects on liver and kidney functions in colon cancer cell (CT-26 cell line) injected tumor model [38].

Taken together, the current study reported for the first time comprehensive effects of* R. verniciflua* on the side effects of chemotherapy, mainly focusing on emesis and immunosuppression. In addition, modulation of 5-HT3 receptor is suggested to be an underlying mechanism of the antiemetic effects of* R. verniciflua*.

## Supplementary Material

Fingerprinting is shown in Supplementary Material, however I think that other part would be fine in test.

## Figures and Tables

**Table 1:** Hematologic parameters and organ weights.

Parameters	Normal	Control	RVX 25	RVX 50	RVX 100	Met 25
WBC (k/*μ*L)	5.44 ± 1.19	3.12 ± 0.45^###^	3.20 ± 0.61	3.87 ± 0.63	5.59 ± 1.62^*∗∗∗*^	4.14 ± 0.28
Neutrophils (k/*μ*L)	0.52 ± 0.12	0.68 ± 0.24	0.62 ± 0.25	0.73 ± 0.18	1.01 ± 0.33	0.93 ± 0.60
Lymphocytes (k/*μ*L)	4.73 ± 1.06	2.27 ± 0.43^###^	2.46 ± 0.48	2.97 ± 0.44^*∗*^	4.33 ± 1.23^*∗∗∗*^	3.06 ± 0.40^*∗*^
Monocytes (k/*μ*L)	0.03 ± 0.02	0.04 ± 0.01	0.04 ± 0.04	0.05 ± 0.02	0.13 ± 0.06^*∗∗∗*^	0.08 ± 0.03
Basophil (k/*μ*L)	0.01 ± 0.00	0.03 ± 0.02^#^	0.02 ± 0.01	0.03 ± 0.01	0.03 ± 0.01	0.03 ± 0.01
Eosinophil (k/*μ*L)	0.05 ± 0.05	0.08 ± 0.06^#^	0.03 ± 0.02	0.09 ± 0.03	0.08 ± 0.04	0.05 ± 0.02

RBC (m/*μ*L)	7.58 ± 0.37	7.09 ± 0.20^#^	6.89 ± 0.67	7.31 ± 0.27	7.31 ± 0.10	7.16 ± 0.23
Hemoglobin (g/dL)	14.68 ± 1.17	14.45 ± 0.71	14.40 ± 1.60	14.75 ± 0.55	15.35 ± 0.73	14.68 ± 0.56
Platelet (k/*μ*L)	1091.50 ± 31.89	907.75 ± 173.30	661.00 ± 302.11	1191.75 ± 330.96^*∗*^	1189.50 ± 46.26^*∗*^	1138.75 ± 139.90

Thymus weight (g) (Relative%)	0.67 ± 0.11	0.34 ± 0.08^###^	0.34 ± 0.05	0.46 ± 0.06^*∗∗*^	0.50 ± 0.03^*∗∗∗*^	0.48 ± 0.06^*∗∗*^
	(0.24 ± 0.04)	(0.15 ± 0.04^###^)	(0.17 ± 0.02)	(0.20 ± 0.02^*∗∗*^)	(0.22 ± 0.01^*∗∗*^)	(0.21 ± 0.04^*∗∗*^)
Spleen weight (g) (Relative%)	0.81 ± 0.10	0.50 ± 0.03^###^	0.47 ± 0.02	0.59 ± 0.10^*∗*^	0.60 ± 0.05^*∗*^	0.58 ± 0.07
	(0.31 ± 0.04)	(0.24 ± 0.02^###^)	(0.22 ± 0.02)	(0.24 ± 0.01)	(0.27 ± 0.02^*∗*^)	(0.25 ± 0.02)

Data are expressed as the mean SD (*n* = 6). ^#^*p* < 0.05 and ^###^*p* < 0.001, compared with normal group; ^*∗*^*p* < 0.05, ^*∗∗*^*p* < 0.01, and ^*∗∗∗*^*p* < 0.001, compared with control group. WBC: white blood cell; RBC: red blood cell.
