# Supplementary material for: Antiemetic and Myeloprotective Effects of Rhus verniciflua Stoke in a Cisplatin-Induced Rat Model
Source: Evid Based Complement Alternat Med. 2017 Feb 8;2017:9830342. doi: 10.1155/2017/9830342 (PMC5320322; doi:10.1155/2017/9830342)
Supplement: Supplementary file 1 — Fingerprinting is shown in Supplementary Material, however I think that other part would be fine in test. [file 9830342.f1.docx]

**Supplementary information**

**Preparation of RVX and fingerprinting analysis.** *Rhus verniciflua* Stoke was obtained from a local farm of *Rhus verniciflua* Stoke in Ok-Cheon (Chung-buk, South Korea). Removed bark from *Rhus verniciflua* Stoke slice (approximately 200 g) was boiled in 2 L of distilled water (DW) for 150 min at 100 °C using a high-speed automatic non-pressure earthen pot (Dae-Woong Pharmacy, Seoul, South Korea), and then the extraction further condensed for 120 min. The extract sample was then centrifuged at 1500 rpm, for 30 min. After centrifuged, the supernatant was filtered using filter paper (150 mesh) twice, and lyophilized using a vacuum-freeze drying system. The final lyophilized extract yield was 0.71%, which was stored at -20 °C for further experiments. To observe the chemical components of RVX, fingerprinting analysis was performed under identical conditions using an 1100 series high-performance liquid chromatography (HPLC) instrument (Agilent Technologies, United States) equipped with an autosampler (G1313A), column oven (G1316A), binary pump (G1312), diode-array detector (G1315B), and degasser (GA1379A). The analytical column, with an Atlantis C18 (4.6 nm × 250 nm; particle size, 5 μm; Waters, Torrance, CA, United States), was kept at 50 °C during the analysis. Data were acquired and processed using ChemStation software (Agilent Technologies, Wilmington, DE, United states). The mobile phase conditions contained 10 % acetonitrile in DW with methanol (A) and 0.1 % formic acid in DW (B). The following solutions were employed: 10 % A and 90 % B over 50 min, and 95 % A and 5 % B to 60 min.

To perform the quantitative analysis, a total five of reference compounds in RVX including protocatechuic acid, fustin, fisetin, sulfuretin and butein (50 μg/mL of 90 % methanol), were obtained, as well as RVX samples (100 μg/mL of 90 % methanol). Histograms were obtained under PDA condition at 254 nm. All of calibration curves of each chemical compound were attained by assessing the peak areas at four concentrations in the range of 2.5 - 200 μg/mL for all of the reference compounds. The linearity of the peak area (*y*) *vs.* the concentration (*x*, μg/mL) curve for each component was used to calculate the contents of the main RVX components.
